# Biometric identification of Black Bengal goat: unique iris pattern matching system vs deep learning approach

**DOI:** 10.5713/ab.22.0157

**Published:** 2022-11-14

**Authors:** Menalsh Laishram, Satyendra Nath Mandal, Avijit Haldar, Shubhajyoti Das, Santanu Bera, Rajarshi Samanta

**Affiliations:** 1 Department of Livestock Production Management, West Bengal University of Animal and Fishery Sciences, Kolkata- 700037, West Bengal, India; 2 Department of Information Technology, Kalyani Government Engineering College, Kalyani, Nadia- 741235, West Bengal, India; 3 ICAR-Agricultural Technology Application Research Institute Kolkata, Indian Council of Agricultural Research, Kolkata, West Bengal 700097, India

**Keywords:** Biometric Identification, Black Bengal Goat, Deep Learning, Goat Identification, Iris Image, Iris Pattern Matching

## Abstract

**Objective:**

Iris pattern recognition system is well developed and practiced in human, however, there is a scarcity of information on application of iris recognition system in animals at the field conditions where the major challenge is to capture a high-quality iris image from a constantly moving non-cooperative animal even when restrained properly. The aim of the study was to validate and identify Black Bengal goat biometrically to improve animal management in its traceability system.

**Methods:**

Forty-nine healthy, disease free, 3 months±6 days old female Black Bengal goats were randomly selected at the farmer’s field. Eye images were captured from the left eye of an individual goat at 3, 6, 9, and 12 months of age using a specialized camera made for human iris scanning. *iGoat* software was used for matching the same individual goats at 3, 6, 9, and 12 months of ages. Resnet152V2 deep learning algorithm was further applied on same image sets to predict matching percentages using only captured eye images without extracting their iris features.

**Results:**

The matching threshold computed within and between goats was 55%. The accuracies of template matching of goats at 3, 6, 9, and 12 months of ages were recorded as 81.63%, 90.24%, 44.44%, and 16.66%, respectively. As the accuracies of matching the goats at 9 and 12 months of ages were low and below the minimum threshold matching percentage, this process of iris pattern matching was not acceptable. The validation accuracies of resnet152V2 deep learning model were found 82.49%, 92.68%, 77.17%, and 87.76% for identification of goat at 3, 6, 9, and 12 months of ages, respectively after training the model.

**Conclusion:**

This study strongly supported that deep learning method using eye images could be used as a signature for biometric identification of an individual goat.

## INTRODUCTION

Of late biometric identification is very vital in this digital era. Animal identification is one of the essential components in traceability. Animal identification facilitates registration of animals covering date of birth, breed information and production record, recording of authorized animal movements, national herd management, payment of appropriate grants, subsidies, insurance claimand a vital tool in animal ownership issue and tracing diseased animals of public and animal health concern [1]. Identification of farm animals is still a challenge at the field level. The traditional methods for animal identification such as ear tagging, branding, and tattooing, toe clipping, ear notching have been widely used but are susceptible to tissue damage, loss or steal [2,3]. Radio frequency identification devices have recently been proposed for traceability purpose [4,5]. But external RF devices are susceptible to theft, tampering, and injury while internal devices are invasive and difficult to maintain. Although DNA based identification system is perfect [6], it requires a long time with the involvement of high cost. Considering the welfare of the animals directly relating with the productivity, the researchers are tempted to use non- invasive, painless biometric method for the identification of the farm animals. The coat pattern of animals is the most recognizable biometric marker. For example, certain body stripes identify zebras and tigers; unique spot patterns recognize cheetahs and African penguins carry etc. Biometrics methods such as retina [7], muzzle [8], face [9] have also been tested in animal identification. The recognition rate of retina, muzzle and face is still unsatisfactory.

Iris recognition may be a new biometric technology for the identification of an individual livestock animal in terms of identification and verification purposes since there is vast pattern variability among different individuals. Daugman [10] was the pioneer in the field of iris recognition. The iris, as an internal (yet externally visible) organ of the eye, remains safe from the environment and remains intact over the time. Iris scanning is a rapid method to capture image digitally. Musgrave and Cambier [11] bagged the patent on system and method of animal identification and animal transaction authorisation using iris patterns. Though iris recognition systems is well developed and already in practice in humans [12,13], it has some inherent problems in animal identification. Unlike human, the animal iris is different in configuration and not circular in shape. It is not possible to use conventional identification techniques to segment, normalise and encode the iris of livestock animal. Capturing a high quality image of iris is one of the major challenges, while the animals are non-corporative and constantly move even when restrained properly at the field conditions. The iris analysis and recognition have been done for cow identification [14,15]. The iris pattern matching has been proposed for recognition of goat recently [16,17]. However, iris pattern matching suggests validation for identification of individual Black Bengal goat at the farmer’s field conditions. There are various deep neural networks that are used earlier for individual identification [18]. Those networks provided the best result for image-based supervised learning techniques. Supervised learning can be defined as a computational technique where the input data is labelled as the desired output. A deep learning-based supervised model is a powerful computational tool for biometric-based individual recognition systems [19,20]. The advantage of deep learning model is to extract features directly from an image. There is no need to extract features individually in a static way for each image. A Convolutional Neural Network (CNN) approach can remove static feature engineering task and extract some important feature which can’t be seen on naked-eye observation in iris images [20]. The feature extraction can be done by a deep neural network using the parameters such as filter size, activation function, max pooling layer etc [21]. Filters help to extract various features from an image according to the filter size. Max pooling layer focuses only on the main features of an image. The activation function can able to omit unnecessary signals from an image to develop a network. The signals are carried by neurons which are main drivers in a deep neural network. A deep learning-based supervised model may be useful for biometric-based individual recognition [18]. Resnet152V2 has been suggested to be the best supervised learning model for gait recognition [22] and emotional differentiation using facial expressions [23]. In the present study, of particular interest was to validate the iris pattern matching by using an artificial intelligence-based deep learning approach for recognition and identification of individual Black Bengal goat (*capra hircus*) at the farmer’s field conditions.

## MATERIALS AND METHODS

### Animal care

The experimental protocol and animal care were met in accordance with the National guidelines for care and use of Agricultural Animals in Agricultural Research and Teaching as approved (approval number: V/PhD/2016/07) by the Ethical Committee for Animal Experiments of West Bengal University of Animal and Fishery Sciences, Kolkata- 700037, West Bengal, India.

### Animals

**Figure 1:**
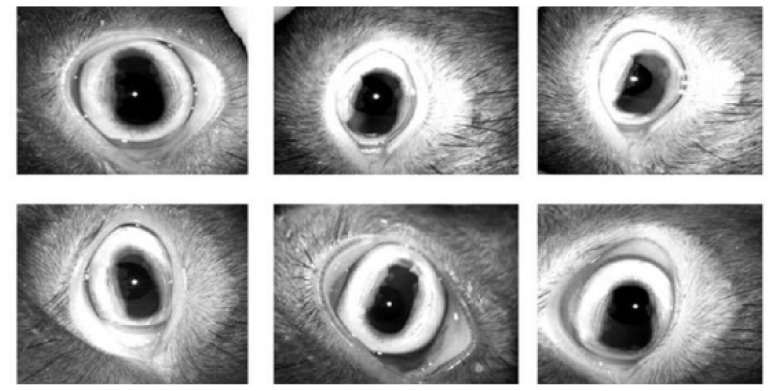
Auto captured iris images of different Black Bengal goats using a specialized iris identification camera IriShield – USB MK2120U which is connected with a light weighted mobile device through the cable for capturing iris images within a distance of 5 cm from the sensor. Eye lid and eye lashes are avoided as much as possible to visualize the whole portion of the iris during capture.

The Black Bengal goat breed was the experimental animal in the present study. The study commenced with the primary visits to identify individual female goats and their owners. Forty nine healthy, disease free, 3 months±6 days old female Black Bengal goats were randomly selected and identified by a neck tag with certain number at Rangabelia under Gosaba Block in Sunderbans delta, West Bengal, India, located at 22°09′55″N 88°48′28″E with an average elevation of 6 metres above the sea level. Data was collected in the form of iris image from the Black Bengal goats at the age of 3 months and then 3 months interval till they attain an age of 12 months. Thus, image of iris was collected at 3, 6, 9, and 12 months of age from each goat. Auto captured iris images of different Black Bengal goats are demonstrated in Figure 1.

### Iris image acquisition

Image acquisition is a process of capturing image which is very important stage to assure the clarity of structure and pattern of iris. The photographic images of the iris were taken using a specialized iris identification camera IriShield – USB MK2120U. The image was captured following the method of Roy et al [17]. The camera had the feature of auto capturing of image. It was operated with an android device. Thus, the camera was connected with a light weighted mobile device through the cable. The inclusion criteria included capturing of iris images within a distance of 5 cm from the sensor. The iris images were captured within a distance of 5 cm from the sensor. The eye-to-camera distance, level of lighting and the amount of reflection were made uniform to reduce the margin of error. As exclusion criteria, eye lid and eye lashes were avoided as much as possible to visualize the whole portion of the iris during capture. The inclusion criteria also included auto capturing of a minimum of 30 images and maximum of 50 images from the left eye of a goat. A total of minimum 5880 iris images (30 images×49 goats×4 occasions) were captured from 49 Black Bengal Goats at 3, 6, 9, and 12 months of age.

### Pre-processing of iris image

Every captured iris image contained some unnecessary parts such as eyelid sclera and pupil. The size of iris image also varied depending on camera to eye distance, illumination level and amount of refection. So, the error was mitigated by cropping the unneeded parts. Images were resized into 700× 650 pixels. Out of 30 to 50 iris images of an individual goat, the best 10 images with maximum coverage of iris area were selected for further processing. Thus, a total of 1960 iris images (10 images×49 goats×4 occasions) were finally processed for matching purpose.

### Iris segmentation

**Figure 2:**
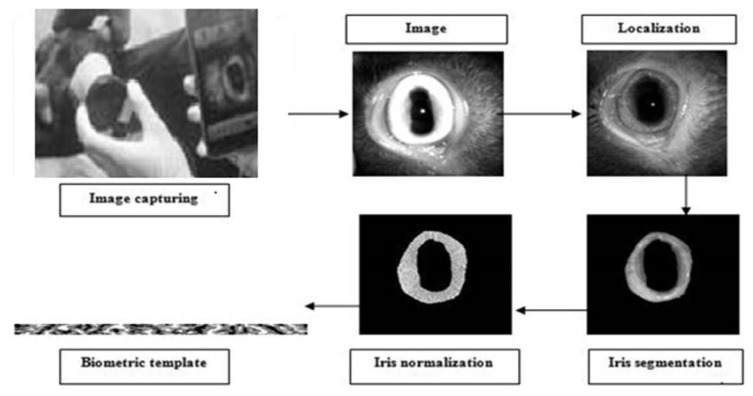
Image capturing, image pre-processing, iris localization and segmentation to locate the near rectangular iris area, iris normalization to remove inconsistency sources and finally generation of iris biometric template of size 480×20 pixels containing some number of bits of information.

Unlike iris of human, goat iris is rectangular in shape and cannot be fitted in any regular shape. Thus, human iris segmentation algorithm would not work to segment iris image of goat eye. Based on the iris segmentation method of Masek [24], a software *iGoat* was developed by Roy et al [17]. The demarcating line between the outer boundary and sclera as well as the boundary line between the inner diameter iris and pupils were done. This helped to locate the near rectangular iris area within the eye as shown in Figure 2. In order to find the boundary of pupil and iris, the gray scale image was converted into binary image with proper threshold value initially. The image was stored into matrix form and started searching from the starting element of the matrix and it continued through the image row wise and was assigned the first non-zero pixel as the starting pixel of the boundary. Through the boundary line the tracing was made in clockwise direction, using Moore neighbourhood approach until the starting pixel reached again.

### Iris normalization

Iris region was normalized in order to remove inconsistency sources like dimensional inconsistencies due to pupil dilation from varying levels of illuminations, varying imaging distance, rotation of camera, head tilt and rotation of the eye at capturing [15]. Normalization yielded same fixed dimension of iris region even after multiple images of same iris under different conditions (Figure 2). The centre of iris region was detected and identified as point and radial vector that passed through the iris region. A total of 240 radial vectors were assigned and 20 data points were selected in each radial vector. For each data point along with each radial line, the Cartesian location was calculated. In the normalized polar representation, intensity values were removed based on the linear interpolation method. In order to compensate the effects of image contrast and illumination, histogram equalization was performed.

### Feature extraction and encoding

Feature encoding was computed convolving the normalized iris pattern with 1D Log Gabor wavelets. The 2D normalized pattern was broken up into a number of 1D signal and then these 1D signals were convolved with 1D Gabor wavelets. The output of filtering was phase quantized to four levels using the method of Daugman [10], with each filter producing two bits of data for each phase. Finally, the encoding process produced a bitwise template of size 480×20 pixels containing some number of bits of information (Figure 2) and a corresponding noise mask which indicated the corrupt areas within the iris pattern, and the bits marked in the template as corrupt.

### Iris pattern matching

Hamming distance (HD) employed by Daugman [10] was used as a metric for iris pattern matching and recognition. HD of two templates was calculated, one template was shifted left or right bit-wise and a number of HD values were calculated from successive shifts for matching. Corrections for misalignments in the normalized iris pattern caused by rotational differences during imaging were also done. When two bits patterns were completely independent, such as iris templates generated from different irises, the HD between the two patterns was equal to 0.5. The independent two bit patterns were totally random, so there was 50 percent probability of setting any bit to 1, and vice-versa. Therefore, half of the bits would agree and half would disagree between the two patterns. When two patterns were derived from the same iris, the HD between them was close to 0 as they were highly correlated. From the calculated HD values, only the lowest was taken, since this determined the best match between two templates. Iris pattern matching was first performed among ten iris images of individual animal at 3, 6, 9, and 12 months of age to authenticate the same animal. The cut off value of 55% was set as minimum threshold matching percentage. Thus, the threshold matching 55% or above signified that the images were from the same Black Bengal goat [17]. Further, iris pattern matching was carried out between animals to distinguish different Black Bengal goats at different times. The threshold matching below 55% between two animals proved that these two Black Bengal goats were different in biometric identity.

### Deep learning analysis

Resnet152V2 deep learning model was applied to process the eye images of goats covering different ages for individual identification. Recently four architectures for training the models: VGG16, ResNet152V2, InceptionV3, and DenseNet201 have been used to classify segments of a sheep using an image dataset of 512 images from 32 sheep for superpixels classification and segmentation of sheep in view of animal tracking and weight prediction [25]. A self-supervised deep learning module has been implemented to accurately recognize Chengdu ma goats [26]. Deep learning based identification model considers an image as input and processes it through different layers, the convolutional layer, the pooling layer, the ReLU activation layer and the fully-connected layer. The model was constructed with various convolution layers, max pooling layers and combination of skip connections. The convolution layer is used to extract the features of an input image in a matrix form. A filter matrix is applied in the convolution layer to extract the features of the input image. The equation of convolution layer as follows.


$$\sum_{a=0}^{m-1}\sum_{\text{b}=0}^{\text{m}-1}\omega_{\text{ab}}{\text{y}}_{\left(\text{i}+\text{a})(\text{j}+\text{b}\right)}^{\text{l}-1}$$

If a m×m filter ω is used, the convolutional layer output will be of size (N−m+1)×(N−m+1). In this case, the contributions (weighted by the filter components) need to be summed up from the previous layer cells.

There is various kind of filter matrix available to extract the features such as edge filter, shape filter etc. Both the filter matrix and convolution layer are depended on the kernel size of a layer. The features are extracted according to the kernel size of a layer. Max pooling layer is used to focus maximum vector of a feature matrix according to their kernel size. It can help to focus most important region of an image. Activation layer is responsible to normalize the information and omit the dead information. Finally, the fully connected layers flatten all information to a 1-D matrix and the information is used to predict the actual output of the input image. The model has number of output terminals same as the number of classes to be classified. In this problem, individual goat has to be identified so that each output terminal can represent identification number of individual goat present in a particular age group.

**Figure 3:**
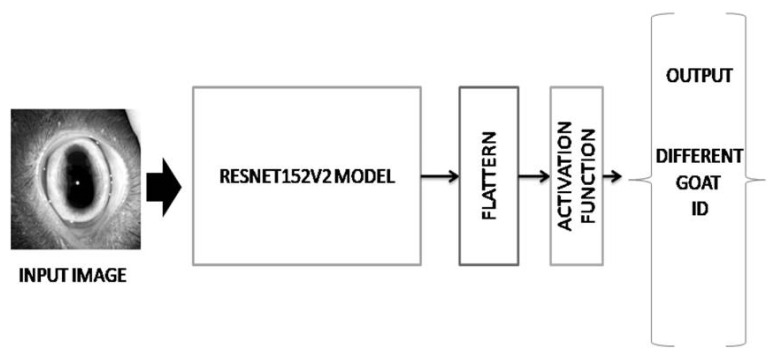
The architecture of the system using Resnet152V2 model demonstrates that the convolution layer is used to extract the features of an input image in a matrix form. A filter matrix is applied in the convolution layer to extract the features of the input image. There are various kind of filter matrix is available to extract the features such as edge filter, shape filter etc. The filter matrix and convolution layer depend on the kernel size matrix of a layer. The features are extracted according to the kernel size of a layer. So the kernel size is responsible for the extracted features from an image. Max pooling layer has been used to focus maximum vector of a feature matrix according to their kernel size. It helps to focus most important region of an image. Activation layer is responsible to normalize the information and omit the dead information. Finally, the fully connected layer flattens all information to a 1-D matrix and the information is used to predict the actual output of the input image.

**Figure 4:**
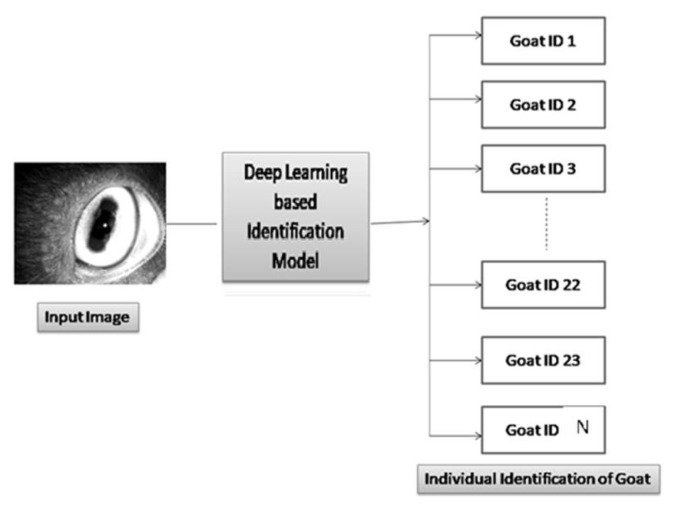
The process of deep learning based identification model demonstrates the verification and identification workflows. An input image is fed into the approached deep learning model and the model processes input eye image of a goat from training set and identify goats with different ID numbers as predicted outputs.

The dataset was created for the training model according to age of individual goat. Figure 1 demonstrated previously how the dataset was created for training the model. Figure 3 demonstrated how the proposed model worked using Resnet152V2 deep learning model. In Figure 4, the model has taken the eye image of a goat from training set and processed it to produce predicted output. An iris pattern consist image was fed into the deep learning model. The deep learning model extracted features from the input image and the extracted features were analysed through the model to find the best matching result from training samples. The high confidence value of one goat was selected as the output result of the model. The output terminals have produced values from 0 to 1. The value of output terminal corresponding to the particular goat has been taken during training of the model and the model has been iterated until the corresponding output terminal has reached above threshold values. The model extracted all features from an image and flattened all information into 1D matrix. The acquired information was subjected to softmax activation function in the last layer for identifying an individual goat ID. The captured eye’s image was divided into 85:15 as training image set and validation image set, respectively. The network was trained with all images from training set and the trained network was tested on test image sets. In test, the unknown eye image has been given to the trained model and the terminal with maximum output value has been selected as predicted output. The predicted goat id has been obtained based on the selected terminal with maximum value.

### Accuracy

Accuracy is one metric for evaluating classification models. Train accuracy (Train acc) is the performance of model that how a model will be learnt based on training dataset. As the output of every training input is known to the model, the weight is adjusted until tolerance of error or maximum epoch is reached. Validation accuracy (Val acc) is the prediction accuracy of the model after training where the output against the input is not known to the model. The prediction is cross-checked by the known result and percentage of the validated actual result is returned. The model performs well if validation accuracy is almost same the training accuracy i.e., the model can predict every unknown input correctly. The following formula was applied to calculate accuracy.


$$Accuracy\;(\%)=\frac{Number\;of\;correct\;Prediction}{Total\;number\;of\;prediction}\times 100$$

## RESULTS

### Iris pattern matching of individual goat over the time using *iGoat* software

Mean iris pattern matching of individual Black Bengal goat covering 49 goats at 3, 6, 9, and 12 month of age is presented in Table 1 (See also Supplementary Table S1). Mean iris pattern matching of individual 49 goats was ≥55% over the time indicating generation of iris templates from the same iris of a particular goat.

### Iris pattern matching between goats at a particular time using *iGoat* software

Mean (±standard error) of iris pattern matching percentages of best images (covering maximum iris without glair) from ten representative goats at 3, 6, 9, and 12 months of age is shown in Table 2 (See also Supplementary Tables S1 to S5). While iris pattern matching was compared between two goats over the time, it was always ≤55% indicating generation of iris templates from different irises of two goats.

### Accuracy of iris pattern matching of goats using *iGoat* software

The overall identification accuracies of iris pattern matching and losses from each age group are presented in Table 3. The accuracies of template matching of goats at 3, 6, 9, and 12 months of ages were recorded as 81.63%, 90.24%, 44.44%, and 16.66%, respectively. The accuracies of iris pattern matching for the goats at 9 and 12 months of ages were low and below the minimum threshold matching percentage.

### Accuracy of eye image matching of goats using Resnet152V2 deep learning model

The accuracies of eye image matching of goats at various ages for individual recognition are shown in Table 4. The maximum accuracies were 95.73% (training) and 92.68% (validation). The best accuracy of eye image matching for individual recognition was recorded at 6 months of age. The lowest accuracy of eye image matching for individual recognition was registered at 9 months of age.

**Figure 5:**
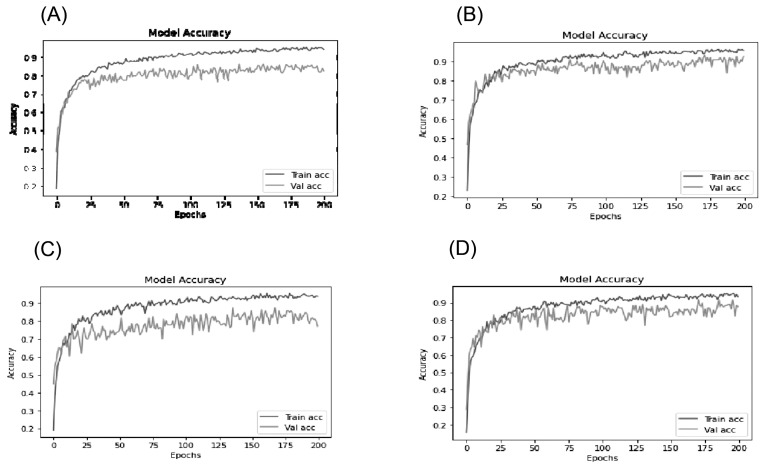
Accuracy of Resnet152V2 deep learning model for 3 months age (A), 6 months age (B), 9 months age (C), and 12 months age (D) demonstrate the accuracy of the model performance which changes during each epoch. For every epoch, the model is being regularized the weight values and prepared the model according to the training dataset. The weight of the model changes with respect to every epoch and thus the accuracy of the model changes accordingly.

The performance analysis of Resnet152V2 deep learning model for different ages is demonstrated in Figure 5 (A–D). The figures show how the learning of the model is changing during each epoch. The learning rate for 6 months of age was the best as compared to learning rates registered for other ages. The graphs demonstrate the accuracy of the final model performance. The X-axis is represented the Epochs and the Y-axis is given the accuracy of the model respectively. Epochs can be defined as number of processes used to learn from the training dataset i.e. how many number of times the learning algorithm works through the whole training dataset. For every epoch, the model is being regularized the weight values and prepared the model according to the training dataset. The weight of the model is changed with respect to every epoch and thus the accuracy of the model is also changed accordingly.

## DISCUSSION

### Iris pattern matching of individual goat over the time

Iris is a thin rectangular structure lies between cornea and the lens of the goat’s eye. Formation of the unique patterns of the iris is random and not related to any genetic factors [27]. As an internal organ (though visible externally) of the eye, the iris is well protected from the environment and remain stable over time and thus the iris pattern remains almost same from birth to death, unless otherwise there is any injury to the iris. Iris architecture is not only complex but also unique to an individual. The iris pattern of each eye is considered as a unique biometric feature [28]. Due to the fact that the two eyes of an individual contain completely independent iris patterns, the iris pattern of left eye of all goats was investigated in the present study. Iris pattern matching and recognition has been reported in goats at a particular age [16,17]. The minimum pattern matching among iris templates of same goat was reported as 59% [17]. In the present study, mean iris pattern matching of 49 Black Bengal goats at 3, 6, 9, and 12 month of age was between 63.71% and 61.45% (Table 1), which was slightly higher than the previous iris pattern matching percentage (59%) in Black Bengal Goats [17]. The present study showed that iris pattern of Black Bengal Goats remained same over the time right from 3 months (kid stage) to 12 months of age (mature stage).

### Iris pattern matching between goats at a particular time

Classification model for identifying different Indian goat breeds has been reported Mandal et al [29]. In earlier studies, iris pattern matching for recognition was proposed based on a small number of goats (≤5) maintained at organized farm and subsequently a limited number of iris image database [16,17]. In the present study, iris pattern matching was performed using 1960 iris image template database to validate the earlier claim in more number of goats and figure out whether this biometric identification technique could be useful at the farmer’s field conditions. The maximum pattern matching among iris templates of different goats was 54% [17]. The present results were comparatively accurate and thus the threshold matching below 55% between two goats over the time suggested that they were different in biometric identity (Table 2).

### Accuracy of iris pattern matching of goats using *iGoat* software

The accuracies of template matching of goats at 3, 6, 9, and 12 months of ages were recorded as 81.63%, 90.24%, 44.44%, and 16.66%, respectively (Table 3). The capture of iris image using auto captured human iris scanner from the goats under uncontrolled situations at the farmer’s field and thus the movement of eye of an individual goat might be the reason for recording the lower accuracies of iris pattern matching for the goats at 9 and 12 months of ages. The gap between matching and mismatching was narrow as the image acquisition was the main challenge in animals due to uncontrolled behaviour. The proper focusing of the image was not possible. The gap could be increased if the goats could be restrained and the iris images could be captured under controlled environment. In the present study, goat iris image was taken using IriShieldTM – USB MK2120U made for capturing human iris image since no iris scanner or specific camera for taking goat iris image was available.

### Accuracy of eye image matching of goats using Resnet152V2 deep learning model

In the present study, the learning rate for Resnet152V2 deep learning model has been used for developing the individual goat identification model based on eye images. The training accuracies and validation accuracies of eye image matching of goats covering different ages ranged between 95.73% and 93.38%, 92.68%, and 77.17%, respectively (Table 4). The result showed that deep learning techniques could be able to recognize individual goat using eye images. After training, the image set of any goat could be identified easily using this deep learning model. Iris texture pattern is determined during fetal development of the eye and unchangeable to age and thus considered as a unique biometric feature [30]. The best accuracy (both training and validation) of eye image matching for individual recognition was recorded at 6 months of age. The chances of over fitting due to less number of dataset availability for 9 months age might be the cause for the lowest accuracy (validation) of eye image matching for individual recognition at 9 months of age. The result of deep learning based eye image matching was more accurate than the iris pattern matching system. The iris pattern recognition has some advantages for exploring more accurate and effective iris feature extraction algorithms under various conditions [31,32]. It was also noted that the iris images of some goats could not satisfy the threshold value at matching using template. However, the deep learning could not allow the threshold value for selection. The deep learning methods, especially the CNN-based methods have accomplished substantial achievement in iris recognition [33,34] and achieved superior performance than the classic iris matching method [35].

## CONCLUSION

The present study suggests that the deep learning-based approach may be able to provide the best accuracies for the biometric identification of Black Bengal goat. It is a non-invasive biometric technique for identification of a goat using automatically captured iris image. Deep learning technique can be implemented as an automated system for individual identification. Deep learning based approach is more cost effective and time effective process than any other processes.

## Figures and Tables

**Table 1:** Iris pattern matching percentage of Black Bengal goats at 3, 6, 9, 12 months of age (n = 49)

Item	Iris pattern matching (%)			

	3 months	6 months	9 months	12 months
Mean	63.71	61.45	62.62	62.49
SD	6.87	4.87	6.52	5.81
SE	0.98	0.70	0.93	0.83

SD, standard deviation; SE, standard error.

**Table 2:** Mean (±standard error) of iris pattern matching percentages of best images from ten goats over 3, 6, 9, 12 months of age

Animal No.	Iris pattern matching (%)									

	P001	P002	P003	P004	P005	P006	P007	P008	P010	P011
P001	100	53.54±0.24	54.22±0.21	54.00±0.39	53.86±0.70	52.99±0.09	53.34±0.19	54.02±0.38	53.34±0.22	54.11±0.45
P002	53.75±0.06	100	54.18± 0.27	54.29±0.21	54.50±0.22	54.07±0.38	53.42±0.58	52.69±0.42	53.68±0.28	53.82±0.64
P003	54.21±0.22	53.71±0.24	100	54.27±0.17	54.15±0.38	53.75±0.63	53.46±0.47	53.68±0.42	54.27±0.27	53.78±0.29
P004	53.52±0.44	53.69±0.40	53.92± 0.19	100	53.77±0.38	53.82±0.69	54.30±0.33	53.96±0.46	53.58±0.34	53.95±0.75
P005	54.28±0.16	54.19±0.37	54.83±0.78	54.41±0.59	100	53.81±0.32	53.98±0.18	53.86±0.40	54.02±0.33	53.82±0.59
P006	53.73±0.49	53.93±0.30	54.52±0.43	53.31±0.65	53.55±0.75	100	53.25±0.66	53.81±0.53	54.29±0.22	53.60±0.26
P007	53.82±0.16	54.17±0.48	54.11±0.50	54.69±0.37	53.76±0.45	54.41±1.05	100	54.62±1.04	53.72±0.43	54.06±0.18
P008	53.37±0.40	53.35±0.36	54.67±0.40	53.33±0.37	54.22±0.36	54.52±0.95	54.32±0.22	100	53.45±0.32	54.89±0.28
P010	53.53±0.29	53.10±0.22	54.20±0.21	53.88±0.28	53.91±0.36	54.27±0.19	53.45±0.28	53.49±0.58	100	53.85±0.44
P011	54.47±0.53	53.58±0.47	54.53±0.30	53.44±0.50	54.96±1.05	53.48±0.42	54.04±0.38	53.59±0.27	53.77±0.43	100

**Table 3:** Overall identification accuracies of iris pattern matching of goats using *iGoat* software

Age (month)	Total number of goats	Number of goats identified	Number of goats not identified	Accuracies (%)
3	49	40	09	81.63
6	41	37	04	90.24
9	36	16	20	44.44
12	36	06	30	16.66

**Table 4:** Overall identification accuracies of eye image matching of goats using Resnet152V2 deep learning model

Age (month)	Training accuracy (%)	Validation accuracy (%)
3	94.08	82.49
6	95.73	92.68
9	93.90	77.17
12	93.38	87.76

## SUPPLEMENTARY MATERIAL

Supplementary Table S1. Iris pattern matching percentage of Black Bengal goats at 3, 6, 9, 12 months of age

Supplementary Table S2. Iris pattern matching percentages of best images from ten goats at 3 month of age

Supplementary Table S3. Iris pattern matching percentages of best images from ten goats at 6 month of age

Supplementary Table S4. Iris pattern matching percentages of best images from ten goats at 9 month of age

Supplementary Table S5. Iris pattern matching percentages of best images from ten goats at 12 month of age

## References

[1] Bowling MB, Pendell DL, Morris DL (2008). Identification and traceability of cattle in selected countries outside of North America. Pro Anim Sci.

[2] Edwards DS, Johnston AM, Pfeiffer DU (2001). A comparison of commonly used ear tags on the ear damage of sheep. Anim Welf.

[3] Gosalvez LF, Santamarina C, Averos X, Hernandez-Jover M, Caja G, Babot D (2007). Traceability of extensively produced Iberian pigs using visual and electronic identification devices from farm to slaughter. J Anim Sci.

[4] Regattieri A, Gamber M, Manzini R (2007). Traceability of food products: general framework and experimental evidence. J Food Eng.

[5] Sahin E, Dallery Y, Gershwin S (2002). Performance evaluation of a traceability system. IEEE T Syst Man Cy B.

[6] Loftus R (2005). Traceability of biotech-derived animals: application of DNA technology. Rev Sci Tech Off Int Epiz.

[7] Allen A, Golden B, Taylor M, Patterson D, Henriksen D, Skuce R (2008). Evaluation of retinal imaging technology for the biometric identification of bovine animals in Northern Ireland. Livest Sci.

[8] Barry B, Gonzales-Barron UA, Mcdonnell K, Butler F, Ward S (2007). Using muzzle pattern recognition as a biometric approach for cattle identification. Trans ASABE.

[9] Corkery GP, Gonzales-Barron UA, Butler F, McDennell K, Ward S (2007). A preliminary investigation on face recognition as a biometric identifier of sheep. Trans ASABE.

[10] Daugman J How iris recognition works.

[11] Musgrave C, Cambier JL (2002). System andmethod of animal identification and animal transaction authorization using iris pattern. US Patent.

[12] Daugman J (2007). New methods in iris recognition. IEEE T Syst Man Cy B.

[13] Feng X, Ding X, Wu Y, Wang PSP (2008). Classifier combination and its application in iris recognition. ntern J Pattern Recognit Artif Intell.

[14] Zhang M, Zhao L An iris localization algorithm based on geometrical features of cow eyes.

[15] Lu Y, He X, Wen Y, Wang PSP (2014). A new cow identification system based on iris analysis and recognition. Int J Biom.

[16] De P, Ghoshal D (2016). Recognition of non circular iris pattern of the goat by structural, statistical and fourier descriptors. Procedia Comput Sci.

[17] Roy S, Dan S, Mukherjee K, Bhattacharjee D, Kole DK, Dey N, Basu S, Plewczynski D (2022). Black Bengal Goat Identification using Iris Images. Pro Int Con Front Com Sys 2020.

[18] Sundararajan K, Woodard DL (2019). Deep learning for biometrics: a survey. ACM Comput Surv (CSUR).

[19] Minaee S, Abdolrashidi A Deepiris: Iris recognition using a deep learning approach. arXiv 2019.1907:09380.

[20] Nguyen K, Fookes C, Ross A, Sridharan S (2017). Iris recognition with off-the-shelf CNN features: A deep learning perspective. IEEE Access.

[21] Nielsen MA (2015). Neural networks and deep learning.

[22] Apostolidis K, Amanatidis P, Papakostas G Performance evaluation of convolutional neural networks for gait recognition.

[23] Hwooi SKW, Loo CK, Sabri AQM (2020). Emotion differentiation based on arousal intensity estimation from facial expressions. Information science and applications.

[24] Masek L (2003). Recognition of human iris patterns for biometric identification [master’s thesis].

[25] Sant'Ana DA, Pache MCB, Martins J (2022). Computer vision system for superpixel classification and segmentation of sheep. Ecol Inform.

[26] Pu J, Yu C, Chen X, Zhang Y, Yang X, Li J (2022). Research on Chengdu Ma goat recognition based on computer vison. Animals.

[27] Wildes RP (1997). Iris recognition: an emerging biometric technology. Pro IEEE.

[28] Prajwala NB, Pushpa NB (2019). Matching of iris pattern using image processing. Int J Recent Technol Eng.

[29] Mandal SN, Ghosh P, Mukherjee K (2020). InceptGI: a convnet-based classification model for identifying goat breeds in India. J Inst Eng India Ser B.

[30] Bowyer KW, Hollingsworth K, Flynn PJ (2008). Image understanding for iris biometrics: a survey. Comput Vis Image Underst.

[31] Benalcazar DP, Zambrano JE, Bastias D, Perez CA, Bowyer KW (2020). A 3D iris scanner from a single image using convolutional neural networks. IEEE Access.

[32] Vyas R, Kanumuri T, Sheoran G, Dubey P (2020). Smartphone based iris recognition through optimized textural representation. Multimed Tools Appl.

[33] Hamd MH, Ahmed SK (2018). Biometric system design for iris recognition using intelligent algorithms. Inter J Educ Mod Comp Sci.

[34] Jayanthi J, Lydia EL, Krishnaraj N, Jayasankar T, Babu RL, Suji RA (2020). An effective deep learning features based integrated framework for iris detection and recognition. J Ambient Intell Humaniz Comput.

[35] Daugman JG (1993). High confidence visual recognition of persons by a test of statistical independence. IEEE Trans Pattern Anal Mach Intell.

