# Supplementary material for: Biometric identification of Black Bengal goat: unique iris pattern matching system vs deep learning approach
Source: Anim Biosci. 2022 Nov 14;36(6):980–9. doi: 10.5713/ab.22.0157 (PMC10164530; doi:10.5713/ab.22.0157)
Supplement: Supplementary Table S1. [file ab-22-0157-Supplementary-Table-1.pdf]

Supplementary Table 1. Iris pattern matching percentage of Black Bengal goats at 3, 6, 9, 12 months of age

Animal No.	Iris pattern matching (%)			
	3 months	6 months	9 months	12 months
**P001**	75.78	55.96	67.66	64.14
**P002**	74.94	60.17	59.94	64.68
**P003**	59.44	63.47	57.02	58.03
**P004**	65.54	64.17	61.21	59.97
**P005**	71.13	59.74	74.98	65.55
**P006**	91.56	78.41	71.45	65.69
**P007**	66.96	56.13	68.23	61.85
**P008**	68.80	64.89	68.28	65.61
**P010**	57.77	55.92	63.90	57.47
**P011**	60.89	64.47	57.43	60.62
**P012**	57.32	64.28	69.75	71.10
**P013**	63.04	69.96	80.58	55.89
**P016**	65.43	65.53	62.77	55.89
**P017**	62.33	59.58	65.59	64.07
**P018**	66.18	59.58	62.99	67.30
**P019**	63.45	60.05	58.45	65.25
**P020**	61.32	58.32	59.58	67.16
**P024**	58.65	56.17	58.12	59.04
**P025**	56.35	58.52	59.41	59.54
**P026**	59.99	56.86	64.34	66.37
**P032**	60.48	65.46	57.14	81.39
**P044**	62.63	56.77	62.76	64.04
**P045**	56.66	66.08	64.10	55.99
**P057**	76.25	73.96	56.21	59.69
**P061**	61.73	66.01	61.41	68.63
**P067**	56.61	61.56	63.86	76.78
**P070**	63.49	62.02	65.76	65.09
**P072**	58.06	71.76	70.60	58.42
**P097**	60.53	58.17	76.27	68.62
**P098**	59.79	55.99	57.15	55.67
**P099**	63.53	64.09	64.88	64.86
**P100**	62.56	61.32	62.99	63.15
**S006**	71.82	57.14	56.97	63.92
**S011**	58.13	67.19	60.63	69.62
**S012**	71.08	61.56	62.47	59.19
**S018**	74.46	61.7	68.73	74.93
**S034**	63.43	57.16	61.27	62.80
**S040**	68.70	62.30	81.26	61.02

**S044**	64.63	60.9	56.12	59.48
**S047**	69.14	58.59	55.60	56.19
**S065**	56.67	61.22	56.47	59.54
**S066**	58.89	56.69	56.12	57.46
**S069**	57.03	57.53	56.48	55.49
**S071**	64.51	58.12	57.11	58.03
**S082**	58.03	59.18	56.32	55.78
**S086**	60.17	60.46	59.46	58.21
**S090**	61.61	58.69	57.05	56.33
**SR408**	58.33	59.53	56.19	57.40
**SR409**	55.82	57.82	55.45	59.05

Supplementary Table 2. Iris pattern matching percentages of best images from ten goats at 3 month of age

**Animal No.**	**Iris pattern matching (%)**									
	**P001**	**P002**	**P003**	**P004**	**P005**	**P006**	**P007**	**P008**	**P010**	**P011**
**P001**	100	52.82	54.8	54.58	51.76	52.71	53.45	54.55	53.76	54.74
**P002**	53.57	100	54.74	53.94	53.88	54.59	54.4	52.86	53.71	54.89
**P003**	54.8	53.11	100	53.99	53.65	54.96	54.5	53.25	54.77	53.08
**P004**	52.66	53.21	53.99	100	52.97	53.59	54.85	54.86	54.54	52.47
**P005**	53.82	53.08	52.97	52.97	100	54.57	54.08	53.99	53.08	52.58
**P006**	52.71	53.89	54.9	53.9	54.74	100	52.73	54.33	53.68	53.83
**P007**	53.42	52.89	54.53	54.35	53.75	54.08	52.93	52.72	53.67	53.66
**P008**	54.53	53.05	54.69	48.89	54.67	54.34	54.83	100	52.76	54.64
**P010**	53.21	52.44	53.9	53.9	53.34	54.4	52.71	51.89	100	53.71
**P011**	52.98	52.99	55.4	53.54	54.57	53.52	54.68	52.87	54.92	100

Supplementary Table 3. Iris pattern matching percentages of best images from ten goats at 6 month of age

**Animal No.**	**Iris pattern matching (%)**									
	**P001**	**P002**	**P003**	**P004**	**P005**	**P006**	**P007**	**P008**	**P010**	**P011**
**P001**	100	53.76	53.76	54.77	54.54	53.08	53.68	53.91	52.76	54.87
**P002**	53.76	100	54.53	54.89	54.94	52.95	51.75	51.68	54.14	52.47
**P003**	53.71	54.28	100	54.76	53.36	52.09	52.75	53.44	53.75	54.5
**P004**	54.77	54.89	53.36	100	54.53	51.95	54.86	54.18	53.02	54.19
**P005**	54.54	54.54	54.76	54.53	100	53.02	53.45	52.74	54.06	53.04
**P006**	53.08	53.08	54.22	51.76	51.76	100	51.68	52.34	54.33	53.26
**P007**	53.68	54.77	52.75	54.86	53.45	53.86	100	54.93	54.95	53.88
**P008**	52.76	52.47	53.75	54.83	53.51	53.65	54.06	100	53.4	54.33
**P010**	52.87	53.22	54.5	54.19	53.63	54.67	53.51	53.48	100	52.69
**P011**	54.92	52.58	54.41	52.05	53.04	52.76	54.34	53.81	53.91	100

Supplementary Table 4. Iris pattern matching percentages of best images from ten goats at 9 month of age

Animal No.	Iris pattern matching (%)									
	**P001**	**P002**	**P003**	**P004**	**P005**	**P006**	**P007**	**P008**	**P010**	**P011**
**P001**	100	53.79	54.17	53.31	54.57	53.09	53.4	54.64	53.61	53.87
**P002**	53.87	100	53.72	54.17	54.58	54.37	53.77	53.69	53.96	52.98
**P003**	54.17	53.72	100	54.17	54.8	53.47	54.01	54.93	54.69	53.79
**P004**	53.33	53.33	54.17	100	54.29	54.86	53.99	54.13	53.53	53.22
**P005**	54.37	54.57	54.8	54.29	100	53.83	54.29	54.11	54.58	54.91
**P006**	54.57	54.37	53.47	54.74	54.83	100	54.68	54.77	54.71	53.09
**P007**	54.09	54.02	54.08	53.91	52.85	52.36	100	53.4	53.04	54.44
**P008**	53.09	53.94	54.54	54.18	54.98	52.87	54.53	100	53.34	54.93
**P010**	54.02	53.37	54.62	53.07	53.71	53.75	54.09	53.93	100	54.76
**P011**	54.54	54.37	54.16	54.43	57.96	52.98	54.19	54.15	53.25	100

Supplementary Table 5. Iris pattern matching percentages of best images from ten goats at 12 month of age

Animal No.	Iris pattern matching (%)									
	**P001**	**P002**	**P003**	**P004**	**P005**	**P006**	**P007**	**P008**	**P010**	**P011**
**P001**	100	53.78	54.16	53.33	54.57	53.09	52.81	52.98	53.23	52.94
**P002**	53.79	100	53.72	54.17	54.58	54.37	53.76	52.52	52.89	54.95
**P003**	54.17	53.72	100	54.17	54.8	54.47	52.57	53.11	53.85	53.75
**P004**	53.33	53.33	54.17	100	53.29	54.86	53.51	52.68	53.21	55.93
**P005**	54.37	54.57	56.8	55.86	100	53.82	54.08	54.61	54.34	54.76
**P006**	54.57	54.37	55.47	52.84	52.85	100	53.89	53.78	54.44	54.23
**P007**	54.09	55.01	55.07	55.62	54.98	57.35	100.00	57.41	53.21	54.25
**P008**	53.09	53.94	55.71	55.4	53.71	57.22	53.87	100	54.31	55.66
**P010**	54.02	53.37	53.79	54.35	54.95	54.27	53.48	54.65	100	54.23
**P011**	55.44	54.37	54.16	53.72	54.25	54.65	52.95	53.54	52.98	100
