# Supplementary material for: Biometric identification of Black Bengal goat: unique iris pattern matching system vs deep learning approach
Source: Anim Biosci. 2022 Nov 14;36(6):980–9. doi: 10.5713/ab.22.0157 (PMC10164530; doi:10.5713/ab.22.0157)
Supplement: Supplementary Table S2. [file ab-22-0157-Supplementary-Table-2.pdf]

**S044**	64.63	60.9	56.12	59.48
**S047**	69.14	58.59	55.60	56.19
**S065**	56.67	61.22	56.47	59.54
**S066**	58.89	56.69	56.12	57.46
**S069**	57.03	57.53	56.48	55.49
**S071**	64.51	58.12	57.11	58.03
**S082**	58.03	59.18	56.32	55.78
**S086**	60.17	60.46	59.46	58.21
**S090**	61.61	58.69	57.05	56.33
**SR408**	58.33	59.53	56.19	57.40
**SR409**	55.82	57.82	55.45	59.05

Supplementary Table 2. Iris pattern matching percentages of best images from ten goats at 3 month of age

**Animal No.**	**Iris pattern matching (%)**									
	**P001**	**P002**	**P003**	**P004**	**P005**	**P006**	**P007**	**P008**	**P010**	**P011**
**P001**	100	52.82	54.8	54.58	51.76	52.71	53.45	54.55	53.76	54.74
**P002**	53.57	100	54.74	53.94	53.88	54.59	54.4	52.86	53.71	54.89
**P003**	54.8	53.11	100	53.99	53.65	54.96	54.5	53.25	54.77	53.08
**P004**	52.66	53.21	53.99	100	52.97	53.59	54.85	54.86	54.54	52.47
**P005**	53.82	53.08	52.97	52.97	100	54.57	54.08	53.99	53.08	52.58
**P006**	52.71	53.89	54.9	53.9	54.74	100	52.73	54.33	53.68	53.83
**P007**	53.42	52.89	54.53	54.35	53.75	54.08	52.93	52.72	53.67	53.66
**P008**	54.53	53.05	54.69	48.89	54.67	54.34	54.83	100	52.76	54.64
**P010**	53.21	52.44	53.9	53.9	53.34	54.4	52.71	51.89	100	53.71
**P011**	52.98	52.99	55.4	53.54	54.57	53.52	54.68	52.87	54.92	100

Supplementary Table 3. Iris pattern matching percentages of best images from ten goats at 6 month of age

**Animal No.**	**Iris pattern matching (%)**									
	**P001**	**P002**	**P003**	**P004**	**P005**	**P006**	**P007**	**P008**	**P010**	**P011**
**P001**	100	53.76	53.76	54.77	54.54	53.08	53.68	53.91	52.76	54.87
**P002**	53.76	100	54.53	54.89	54.94	52.95	51.75	51.68	54.14	52.47
**P003**	53.71	54.28	100	54.76	53.36	52.09	52.75	53.44	53.75	54.5
**P004**	54.77	54.89	53.36	100	54.53	51.95	54.86	54.18	53.02	54.19
**P005**	54.54	54.54	54.76	54.53	100	53.02	53.45	52.74	54.06	53.04
**P006**	53.08	53.08	54.22	51.76	51.76	100	51.68	52.34	54.33	53.26
**P007**	53.68	54.77	52.75	54.86	53.45	53.86	100	54.93	54.95	53.88
**P008**	52.76	52.47	53.75	54.83	53.51	53.65	54.06	100	53.4	54.33
**P010**	52.87	53.22	54.5	54.19	53.63	54.67	53.51	53.48	100	52.69
**P011**	54.92	52.58	54.41	52.05	53.04	52.76	54.34	53.81	53.91	100
