# Supplementary material for: Biometric identification of Black Bengal goat: unique iris pattern matching system vs deep learning approach
Source: Anim Biosci. 2022 Nov 14;36(6):980–9. doi: 10.5713/ab.22.0157 (PMC10164530; doi:10.5713/ab.22.0157)
Supplement: Supplementary Table S4. [file ab-22-0157-Supplementary-Table-4.pdf]

Supplementary Table 4. Iris pattern matching percentages of best images from ten goats at 9 month of age

Animal No.	Iris pattern matching (%)									
	**P001**	**P002**	**P003**	**P004**	**P005**	**P006**	**P007**	**P008**	**P010**	**P011**
**P001**	100	53.79	54.17	53.31	54.57	53.09	53.4	54.64	53.61	53.87
**P002**	53.87	100	53.72	54.17	54.58	54.37	53.77	53.69	53.96	52.98
**P003**	54.17	53.72	100	54.17	54.8	53.47	54.01	54.93	54.69	53.79
**P004**	53.33	53.33	54.17	100	54.29	54.86	53.99	54.13	53.53	53.22
**P005**	54.37	54.57	54.8	54.29	100	53.83	54.29	54.11	54.58	54.91
**P006**	54.57	54.37	53.47	54.74	54.83	100	54.68	54.77	54.71	53.09
**P007**	54.09	54.02	54.08	53.91	52.85	52.36	100	53.4	53.04	54.44
**P008**	53.09	53.94	54.54	54.18	54.98	52.87	54.53	100	53.34	54.93
**P010**	54.02	53.37	54.62	53.07	53.71	53.75	54.09	53.93	100	54.76
**P011**	54.54	54.37	54.16	54.43	57.96	52.98	54.19	54.15	53.25	100

Supplementary Table 5. Iris pattern matching percentages of best images from ten goats at 12 month of age

Animal No.	Iris pattern matching (%)									
	**P001**	**P002**	**P003**	**P004**	**P005**	**P006**	**P007**	**P008**	**P010**	**P011**
**P001**	100	53.78	54.16	53.33	54.57	53.09	52.81	52.98	53.23	52.94
**P002**	53.79	100	53.72	54.17	54.58	54.37	53.76	52.52	52.89	54.95
**P003**	54.17	53.72	100	54.17	54.8	54.47	52.57	53.11	53.85	53.75
**P004**	53.33	53.33	54.17	100	53.29	54.86	53.51	52.68	53.21	55.93
**P005**	54.37	54.57	56.8	55.86	100	53.82	54.08	54.61	54.34	54.76
**P006**	54.57	54.37	55.47	52.84	52.85	100	53.89	53.78	54.44	54.23
**P007**	54.09	55.01	55.07	55.62	54.98	57.35	100.00	57.41	53.21	54.25
**P008**	53.09	53.94	55.71	55.4	53.71	57.22	53.87	100	54.31	55.66
**P010**	54.02	53.37	53.79	54.35	54.95	54.27	53.48	54.65	100	54.23
**P011**	55.44	54.37	54.16	53.72	54.25	54.65	52.95	53.54	52.98	100
